# Loop Closing Detection in RGB-D SLAM Combining Appearance and Geometric Constraints

**DOI:** 10.3390/s150614639

**Published:** 2015-06-19

**Authors:** Heng Zhang, Yanli Liu, Jindong Tan

**Affiliations:** 1 School of Information Engineering, East China Jiaotong University, Nanchang 330013, China;; 2 Department of Mechanical, Aerospace and Biomedical Engineering, University of Tennessee, Knoxville, TN 37996, USA; E-Mails: hzhang69@utk.edu (H.Z.); tan@utk.edu (J.T.)

**Keywords:** SLAM, binary descriptor, geometric constraints, hierarchical clustering

## Abstract

A kind of multi feature points matching algorithm fusing local geometric constraints is proposed for the purpose of quickly loop closing detection in RGB-D Simultaneous Localization and Mapping (SLAM). The visual feature is encoded with BRAND (binary robust appearance and normals descriptor), which efficiently combines appearance and geometric shape information from RGB-D images. Furthermore, the feature descriptors are stored using the Locality-Sensitive-Hashing (LSH) technique and hierarchical clustering trees are used to search for these binary features. Finally, the algorithm for matching of multi feature points using local geometric constraints is provided, which can effectively reject the possible false closure hypotheses. We demonstrate the efficiency of our algorithms by real-time RGB-D SLAM with loop closing detection in indoor image sequences taken with a handheld Kinect camera and comparative experiments using other algorithms in RTAB-Map dealing with a benchmark dataset.

## 1. Introduction

Loop closing detection [1,2] consists of determining whether the robot is in the area that has been visited before according to current sensor information, or whether the current position of the robot has been described in the map. In other words, the robot can observe and accurately judge the landmarks or scenes observed before. Loop closing detection is an effective way to eliminate errors and improve the accuracy of the robot localization and mapping [3,4].

**Figure 1:**
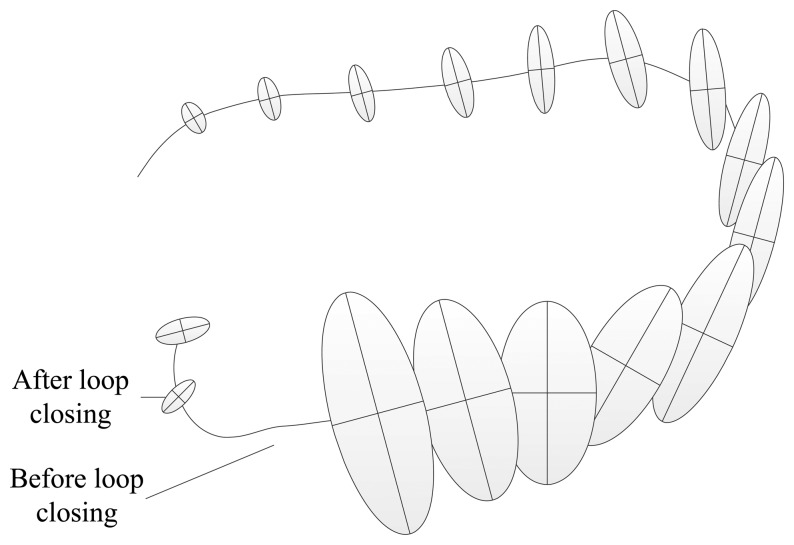
The diagram of loop closing. There is a certain degree of error in the observation of each step. When the robot (or the camera) observes the area that have been seen in the past, a new constraint can be added to correct a series of poses. This may be repeated several times. With these new constraints, the accumulated error of map building and localization can be considerably reduced.

Due to error accumulation, pose estimation for prior landmarks observed by the robot is more accurate than for posterior landmarks [5]. Therefore, when a loop closure is formed, the robot can use accurate landmarks to correct inaccurate landmarks. Figure 1 shows the diagram of loop closing. From Figure 1, we can see that the errors and uncertainties of the robot pose increase rapidly with the robot's movement, but the errors and uncertainties may decrease rapidly when the robot returns to the position which has been visited and re-observes some landmarks.

The right loop closing can be used to correct the errors of the odometer, and thus be helpful to obtain consistent map. However, wrong loop closing will disturb the following pose estimates, and may even completely destroy the existing map, which is unexpected in RGB-D SLAM. Loop closing is a difficult problem in unknown environment map building. Currently there are three issues: perceived ambiguity, large-scale data and evaluation requirements.

(1) Perceived ambiguity: similar observations are not necessarily from the same scene, which accordingly cause false judgment on loop closing. There are two reasons. One is that the sensor obtains partial information of the environment and another reason is that there are many similar objects in both indoor and outdoor environment like similar desks, chairs, air-conditions, walls, buildings and so on.
(2) Large-scale data: when loop closing is detected, the current observation data is compared with the previous data to calculate their similarity or estimate the probability that they are from the same location. The size of the data is growing with the runtime or the number of visited places. It can be scaled up to tens of thousands of frames.
(3) Evaluation requirements: loop closing detection can add new constraints to reduce or even eliminate cumulative error in the incremental estimation. However, inaccurate loop closing may have an impact on the results and lead to wrong convergence when optimizing. Therefore, correctness of judgment is the key to loop closing detection. The index used to evaluate the loop closing detection is Precision-Recall curve. Precision is defined as the number of correct loop closings detected over the number of total loop closings. Recall is defined as the number of correct loop closings detected over the number of true loop closings. Requirement for precision is very high [6], which is close to 100%. Recall is not too low, otherwise, loop closing information is not used to effectively correct the trajectory.

In this work, we use fast matching algorithm fusing local geometric constraints and multi-feature points to detect loop closing. In the algorithm, we make use of binary descriptors to find appropriate matching pairs and combine geometric constraints to determine the correct matching of feature points. Feature point appearance integrated with local geometric relation reduces perceived ambiguity as much as possible, since there are many similar objects in scene but with different geometric constraints. In the search process, the binary descriptor is stored by locality sensitive hashing table and hierarchical clustering tree is adopted to search binary features. In the process of feature point matching, hamming distance instead of Euclidean distance is employed to compute the distance between points, which can deal with large-scale data and improve the speed and accuracy of calculating similarity.

This article addresses the problem of loop closing in RGB-SLAM, and has three main contributions: (1) We adopt binary descriptors as visual features inspired by the related works [7–9], which have advantages in accuracy, processing time and memory consumption since they combine intensity and geometric information to improve the ability of faster and accurate matching; (2) We store binary descriptors by Locality-Sensitive-Hashing (LSH) technique [10] and use hierarchical clustering trees [11] to search binary features, which improve the accuracy and speed of matching; (3) Appearance-only matches may cause false judgment on loop closing because of the similar objects in the scene. The algorithm in the paper integrates appearance with local geometric constraints to improve accuracy for the judgment on loop closing.

This paper is organized as follows. In Section 2 a brief survey of related work is presented. Then the BRAND (binary robust appearance and normal) descriptor is described in Section 3. In Section 4 and Section 5, Locality-Sensitive Hashing technique and hierarchical clustering based searching algorithm are introduced respectively. Section 6 details the matching algorithm integrated appearance with local geometric constraints. Some experimental results are presented in Section 7 and, finally, we provide some conclusions and future work.

## 2. Related Work

Loop closing is now considered as one of the main challenges in the real-time SLAM system, getting more and more recognition and attraction. In SLAM, the problem of loop closing is divided into two parts: the first part is correctly judging whether a robot has returned to a previously visited location and the next part is how to correct the errors of a map. However, the two parts can not be completely separated in practical applications.

Many algorithms are based on the image matching on comparing them using the bag of words approach [12]. A bag of words (BoW) is a structure that allows representing an image, which makes it possible to perform comparisons with thousands of images. On the foundation of BoW, Nister *et al.* [13] put forward tree-based storage and management to improve the retrieval efficiency significantly. Schindler *et al.* [14] analyze feature selection problem in the words construction and utilize information gain to assess feature. They only select distinguishable features to construct words, so the performance and scalability of the algorithm are significantly improved. Cummins *et al.* [15] have chosen to use Chow Liu tree to approximatively describe the correlation between words and makes full use of contextual information to reduce perceptual aliasing. In [16], Cummins *et al.* demonstrate the appearance-based SLAM for very large scale place recognition with FAB-MAP 2.0. Thomas *et al.* [17] integrated the BoW place recognition system into their extended KinectFusion framework to provide visual loop closure detection.

Calculating the distance between two poses is the most intuitive way to judge if two poses are in the same scene. If the distance is small enough, loop closing may be formed. But because of cumulative error, after long-time movement, there will still be a large deviation between estimated poses even if the robot is in the same position. Bosse *et al.* [18] use Dijkstra shortest path algorithm to calculate minimal uncertainty path. Considering the sensor measuring range, Olson *et al.* [19] use Mahalanobis distance between the poses to determine the likelihood of the observation coincident. But there are some limitations in the method. When the actual error is greater than the estimation error, right loop closing information will be ignored.

Appearance based approaches are used to loop closing detection obtained with promising results Newman *et al.* [20] should not rely on using the same source of geometric measurements for mapping and localization to ensure the robustness of loop closing information. They put forward saliency feature to judge loop closing. Evaluation criteria will be varied with different representation. For binary feature description, Sunderhauf *et al.* [21] calculate hamming distance between features to measure similarity of the observations. For the representation with bag of words, the traditional method is that TF-IDF (term frequency–inverse document frequency) is first calculated weight and then the cosine similarity method is used to solve the problem. Cummins *et al.* [15] establish the probability of an observation model for each location and utilize Bayesian framework to compute the probability that the indistinctive observations come from the same place.

Relocalization lies in locating the robot on an existing map when its pose is unknown. Relocalization [22,23] is conventionally a crucial component in loop-closing detection. Williams *et al.* [24] use the filter approach to perform relocalization and loop closing, which solves some problems encountered by previous monocular SLAM systems-tracking failure, map merging, and loop closing detection. In order to find connections from the current view to other corresponding views in the map, Konolige *et al.* [25] use a vocabulary tree to propose candidate views, and a geometric filter to eliminate false positives. Eade and Drummond [26] unify relocalization and loop closing in a graph-based RTMS (Real Time Monocular SLAM) system, using both appearance and structure to confirm likely matches. They implement a bag of word approach for ranking potential loop closings. Pirker *et al.* [27] use a keyframe-based SLAM framework to perform relocalization and loop closing for the operation over long periods of time indoors and outdoors. They adopt the graph structure to speed up loop closing correction. Tan *et al.* [28] employ SIFT features with key frame representation to perform global localization on monocular SLAM in dynamic environment. Mur-Artal *et al.* [29] use a bag of words with ORB features for both relocalization and loop closing. They present the keyframe-based SLAM that can deal with severe viewpoint change. Unlike visual SLAM approaches that use keyframes, Johannsson *et al.* [30] use a bag of words approach to detect loops in the reduced pose graph.

Our system is the improvement on appearance based approaches. The algorithm concentrates on multi feature points matching in RGB-D images. The matching algorithm combines appearance information with geometric relation of feature point. RGB-D sensor provides rich 3D spatial information and color texture information for the matching. In the experiments, we compare our method with appearance based approaches. Our algorithm shows better performance than appearance based method.

## 3. BRAND Descriptor

This section describes the binary descriptor that we have employed in our work. There are many feature descriptor methods, which are mainly divided into two categories: gradient histogram-based feature descriptors like SIFT [31], SURF [32] and binary feature descriptors like BRISK [7], ORB [33], and BRIEF [8]. In SURF and SIFT, since matching between the vectors is usually performed by computing the squared distance between the vectors, they present high computational cost and memory consumption when matching thousands of features. Binary feature descriptors are described with a binary string. These descriptors are computed by pairwise intensity comparison tests, using simple intensity difference tests, which have characteristics of less memory consumption, faster processing in creation and matching process. The distance between two binary strings can be measured using the Hamming distance. The Hamming distance equation is given as:

(1) $$\Delta_{\text{hamming}}\left(x,y\right)=\sum_{i=1}^{n}x_{i}⊕y_{i}=\sum_{i=1}^{n}b\left(x_{i},y_{i}\right)$$

where *b*(*x*, *y*) represents bit inequality, and *x_i_* and *y_i_* are the i-th bits in the descriptors *x* and *y*, respectively.

(2) $$b\left(x,y\right)=\{\begin{array}{cc} 1 & x\neq y\\ 0 & x=y \end{array}$$

In this work, we adopt binary robust appearance and normal (BRAND) descriptor [9], which combines appearance and geometric shape information from RGB-D images. BRAND has advantages in accuracy, processing time and memory consumption since it combines intensity and geometric information to improve the ability of fast and accurate matching. It is invariant to rotation and scale transform and suitable for applications with low memory consumption and high speed. In this work, we chose to patch P of size *S* × *S*(*9* < *S* < 48) and the patch where the set of pixel pairs (**x***_i_*, **y***_i_*) ∈ *P* are indicated with line segments. For decreasing the sensitivity to noise and increasing the stability in the pixels comparison, we pre-smooth the patch with a Gaussian kernel.

## 4. Features Searching Based on Locality-Sensitive Hashing

### 4.1. Locality-Sensitive Hashing

In many application fields, we face and have to handle data which is massive and has very high dimension. How to find one or more data most similar or closet to a certain data is a difficulty. If it is a low-dimensional data set, we adopt a linear search to solve the problem. However, if we use linear search for a mass of high-dimensional data, it will be very time-consuming. The method of NNS (Nearest Neighbor Search) is a good way to solve the problem. There are many methods proposed like K-D (K-Dimension) tree [34,35], and ANN (Approximate Nearest Neighbor), *etc.* Locality-Sensitive Hashing (LSH) and its variants [36,37] are well-known methods for solving the c-approximate NNS problem in high-dimensional space. Indyk *et al.* [36] firstly used the LSH scheme in binary Hamming space {0, 1}*^d^* and Datar *et al.* [37] extended it to Euclidean space R*^d^*. The method of LSH has wide applications in various fields, such as text classification, image retrieval, fingerprint matching, computational biology and so on. These applications are required to calculate the similarity between a large amount of data (or distance), the use of LSH will accelerate the matching speed.

**Figure 2:**
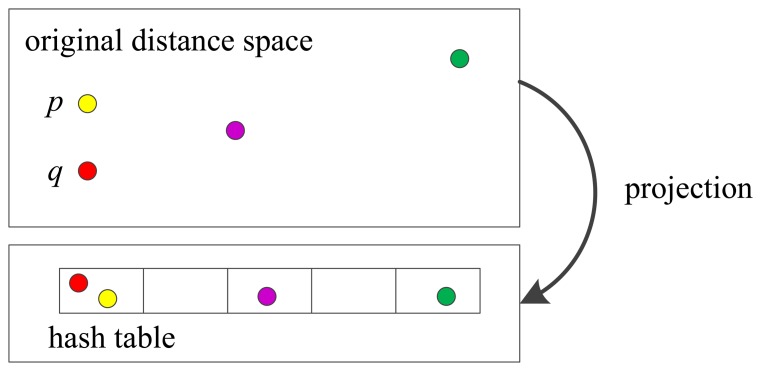
The schematic diagram of LSH.

As shown in Figure 2, the idea of LSH is to hash the points in a way that the probability of collision is much higher for points which are close to each other than for those which are far apart. In Figure 2, the point *p* and *q* are close in the original space, so they are projected into the same bin. The core of LSH algorithm is to construct a set of hash functions that keep the relativity of distance and use the function to classify the similar data into the same hash bucket. In this work, the similarity measure is the Hamming distance, so the hashing function is a subset of bits of the binary number. Similar features have greater possibility to be fallen into the same bucket. When matching, feature points in the same bucket with matching points are considered as a candidate set. Consequently, a large number of features which are not in the same bucket are excluded.

### 4.2. Hash Tables for Binary Descriptors

It is a natural choice that LSH strategy is used to organize all the binary descriptors into a set of hash table. Because binary descriptors are already in Hamming space, we can easily construct a hash function to map similar binary descriptors into the same bucket. Since the similarity measure is the Hamming distance, a hash function becomes a set of binary bit string, but here we use fewer tables. It guarantees that those descriptors with the same subset of bits are stored in the same bucket. The advantage is that assignment of binary descriptors in the bucket has a uniform distribution due to the high difference in the descriptor's bits. It is suitable because a uniform distribution in the buckets helps to approximately maintain a constant time when accessing to the descriptors in the buckets.

The 3D world position of the interest 3D point is stored in the map and attached to its binary descriptor. So, when a binary descriptor is retrieved from arbitrary binary hash tables, we will have access to its 3D position.

In the process of environmental detection, each binary descriptor b*_i_* is passed to the hash function and can access to the corresponding buckets in the hash tables where matches for b*_i_*. We adopt a linear NN search in all the retrieved binary descriptors from the buckets searching for the descriptor that minimizes the Hamming distance with b*_i_*. Assuming b*_i_m__* is the best match for bi, and assuming p*_i_m__* is the 3D position of the best binary match b*_i_m__*. A set of 2D-3D pairs is represented as follows:

(3) $$C=C\cup \left\{({\text{s}}_{i},{\text{p}}_{i_{m}})\right\}$$

If |*C*| > *c*_size_ then it is passed to the pose estimation module. We use a three-point pose estimator plus RANSAC to estimate pose. If the pose estimator finds a minimum set of inliers in *C*, it is considered successful and is passed to loop closing adjustment process.

## 5. Binary Features Searching Based on Hierarchical Clustering

### 5.1. Batch Clustering Tree Building

Suppose features dataset is *D* = {**p***_i_*|1 ≤ *i* ≤ *N*}, tree building process starts with all the points in the dataset and divides them into *K* clusters, which are respectively represented with *C*_1_, *C*_2_, …, *C_K_*, where *K* is called the branching factor and a parameter of the algorithm. The basic process of the algorithm is that firstly we choose *K* points {**q**_1_, **q**_2_, ⋯ , **q***_K_*} at random from *D* as the center of cluster *C*_1_, *C*_2_, …, *C_K_*, and then assign other points to each center the points closer to that center than to any of the other centers, the equation is as follow:

(4) $$\begin{array}{ccccc} {\text{p}}_{i}\in C_{i} & \text{iff} & \tilde{{\text{p}}_{i}{\text{q}}_{j}}\leq \tilde{p_{i}{\text{q}}_{k}}, & 1\leq i\leq N, & 1\leq k\leq K \end{array}$$

where $\tilde{{\text{p}}_{i}{\text{q}}_{j}}$ is similarity distance between two feature points p*_i_* and q*_j_*. Because we adopt binary feature descriptor, $\tilde{{\text{p}}_{i}{\text{q}}_{j}}$is hamming distance. The batch tree building process is described in Algorithm 1.

The clustering tree building process (presented in Algorithm 1) begins with all the points in features dataset *D* and divides them into *K* clusters, where *K* is called the branching factor. *K* points are selected at random as the cluster centers. The algorithm is repeated recursively for each of the resulting clusters until the number of points in each cluster is below a certain threshold *F_L_*.

We simply select centers of the cluster randomly from the input points, which results in a much simpler and more efficient algorithm for tree building. Moreover, independence between clusters helps us to build multiple trees and conducts parallel search when matching. Since we select the cluster centers randomly, the algorithm that we use multiple randomized trees is effective. However, when the closest neighbor to the query point lies just across a boundary from the domain explored, matching is likely to fail. To avoid the situation, we can explore them in parallel because the closest neighbor may well lie in different domains in different trees.


**Algorithm 1** One hierarchical clustering tree building
**Input:**
*D*: features dataset; *N:* size of *D*; *K*: branching factor; *F_L_*: maximum leaf size**Output:**
*T*: clustering tree1:generate head node of the tree *T*, represented with *header*2:**if**
*N* < *F_L_*
**then**3:expand all points in *D* as child nodes of *header*4:**else**5: we choose *K* points {q_1_, q_2_, ⋯ , q*_K_*} randomly from *D*, *K* clusters *C* = {*C*_1_, *C*_2_, ⋯ , *C_K_*}, q*_k_* is the center of *C_k_*6: **for**
*i* = 1 → *N*
**do**7:  *min*_*length* = *MAX*_*NUMBER*, *k* = 08:  **for**
*j* = 1 → *K*
**do**9:   **if**
$\tilde{{\text{p}}_{i}{\text{q}}_{j}}\leq \min\_\text{length}$
**then**10:     
$\min\_\text{length}=\tilde{{\text{p}}_{i}{\text{q}}_{j}}$, *k* = *j*11:   **end if**12:  **end for**13: **end for**14: **for**
*i* = 1 → *K*
**do**15:  recursively apply the algorithm to the points in *C_i_*16: **end for**17:**end if**


The search procedure of parallel hierarchical clustering tree is presented in Algorithm 2. The algorithm begins with a single traverse of each tree, during which the node closest to the query point is picked and then explored recursively, at the same time the unexplored nodes are added to a priority queue. When the leaf node is reached, we search all the points contained within the node. After all the trees have been searched once, we select from the priority queue the closest node to the query point to restart the traverse of tree. In order to control the execution time of the algorithm, the search ends when the number of points examined exceeds the given value.


**Algorithm 2** Search algorithm of parallel hierarchical clustering tree
**Input:**
*T* = {*T_i_*|*i* = 1, 2,⋯, *M*}: hierarchical clustering trees;1:*Q*: query point;2:*L_max_*: maximum leaf size**Output:**
*K* nearest approximate neighbors of query point *Q*3:*L* = 0 ⊲ *L* is the number of points searched4:generate two empty priority queues *Pf* and *Ps*5:for *i* = 1 → *M* do6: TraverseTree(*T_i_*, *Pf*, *Ps*)7:**end for**8:**while**
*Pf* is not empty and *L* < *L*_max_
**do**9: *N* = top of *Pf*10: TraverseTree(*N*, *Pf*, *Ps*)11:**end while**12:13:**function** TraverseTree(*N*, *Pf*, *Ps*)14: **if** node *N* is a leaf node **then**15:  Search all the points in *N* and add them to *Ps*16:  *L* = *L* + |N|17: **else**18:  *C* = child nodes of *N*19:  *C_q_* = closest node of *C* to query *Q*20:  *C_p_* = *C*\*C_q_* ⊲ delete *C_q_* from *C*21:  Add all nodes in *C_p_* to *Pf*22:  TraverseTree(*C_q_*, *Pf*, *Ps*)23: **end if**24:**end function**


### 5.2. Incremental Clustering Tree Building

Compared to the linear search, batch clustering tree building has improved the efficiency of the algorithm, but cannot meet real requirements in the RGB-D simultaneous localization and mapping for mobile robot. With the increase of the map, the search efficiency is dramatically reduced. Therefore, we design the algorithm of incremental clustering tree building (presented in Algorithm 3) and new feature points are merged into previous clustering tree than clustering tree rebuilding. The algorithm uses previous results to accelerate the clustering process and improve its efficiency.

In the algorithm, we use dual cycle to find the minimum cluster distance and judge new data according to minimum distance: (1) if the distances between new point and every cluster center are all greater than minimum cluster distance, the new point will not belong to any cluster and a new cluster is built; (2) if the distance between new point and only one cluster is less than minimum cluster distance, the new point will belong to the cluster; (3) if the distances between new point and some cluster centers are all less than minimum cluster distance, these clusters are merged into a new cluster and the new point is added to the new cluster.


**Algorithm 3** Incremental clustering tree building
**Input:**
*p*: feature point; *K*: branching factor**Output:**
*T′*: Incremental clustering tree1:select one point from *C*_i_ as central point randomly, compose the central point set {*t*_1_, *t*_2_, ,⋯ *t_K_*}2:
$\min\_\text{length}=\tilde{t_{1}t_{2}}$ ⊲ *min_length*: minimum cluster distance3:**for**
*i* = 1 → *K* − 1 **do**4: **for**
*j* = *i* + 1 → *K*
**do**5:  **if**
$\tilde{t_{i}t_{j}}<\min\_\text{length}$
**then**6:   
$\min\_\text{length}=\tilde{t_{i}t_{j}}$;7:  **end if**8: **end for**9:**end for**10:
$\min\_\text{length}1=\tilde{\text{p}t_{1}}$, *k* = 1, *count* = 0 ⊲ *min_length*1: minimum distance between *p* and clusters11:**for**
*i* = 2 *− K*
**do**12: **if**
$\tilde{\text{p}t_{i}}<\min\_\text{length}1$
**then**13:   
$\min\_\text{length}=\tilde{\text{p}t_{i}}$, *k* = *i*14: **end if**15: **if**
$\tilde{\text{p}t_{i}}\leq \min\_\text{length}$
**then**16:  *count* + +, *D* (*count*) = *i* ⊲ the number of clusters less than*min_length* and number of cluster17:**end if**18:**end for**19:**if**
*min_length*1 > *min_length*
**then** ⊲ create a new cluster20: *C_K_*_+1_ = {p}, *K* = *K* + 121:**else if**
*count* == 1 **then**22: *C_k_* = *C_k_* ∪ {p} ⊲ add to the *k* – *th* cluster23:**else**24: **for**
*i* = 1 → *count*
**do** ⊲ combine clusters25:  *C_k_* = *C_k_* ∪ *C_D_*_(_*_i_*_)_, *K* = *K* − 126: **end for**27: *C_k_* = *C_k_* ∪ {p} ⊲ add to the merged cluster28:**end if**


## 6. Multi Feature Points Matching Algorithm Fusing Local Geometric Constraints

### 6.1. Introduction of the Algorithm

**Figure 3:**
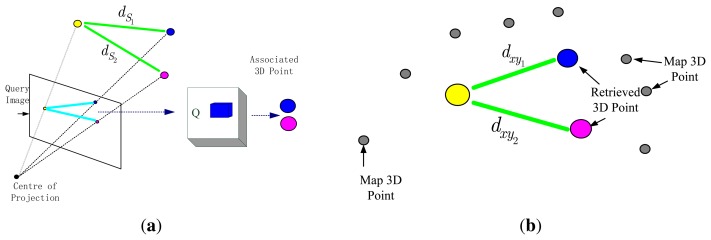
Illustration for the matching of multi feature points using local geometric constraints. (**a**) For each one of the two salient points, 3D point relative to the camera coordinate system is computed using the depth information, and then 3D distance *d_S_i__* in between salient points can be calculated; (**b**) The binary descriptors are computed for two salient points in the query image, these descriptors are used to retrieve best binary match from the hash table Q. The distance *d_xy_i__* in between pairs of 3D points in the world (map points) can be calculated.

The core idea of the algorithm is multi feature point matching method fusing local geometric constraints. We use LSH technique to store feature points in Hash table and adopt hierarchical clustering to fast binary search. Given an image, salient points in the image are extracted using the FAST corner detector [38]. The main idea is shown in Figure 3. First we calculate the binary descriptors for two salient points in the query image, next use these binary descriptors to retrieve their the best match in the hash table Q, then compute distance *d_xy_* between these points in the world coordinate system, which are associated to the descriptors retrieved from the hash table Q. On the other side, we use the depth information to compute the distance between these two 3D points, the distance is called *d_S_* and two 3D points are relative to the camera coordinate system. If the difference in between *d_S_* and *d_xy_* is less than a threshold Δ*_D_*, we find the best matches. Otherwise, we will discard them (at least there is a point to be replaced by another point). Finally, if the total number of selected matches exceeds a predetermined size, we will have to carry out pose estimation.

### 6.2. Multi Feature Points Matching Algorithm

Let f′*_i_* is the *i*-th element of the feature point set F′ which detected from the current image frame, and b′*_i_* is its corresponding binary descriptor. We search for the best matching f*_i_m__* in map feature point set F and construct the set of pairs *M* = {(f′*_i_*, f*_i_m__*)|*i* = 1, 2, …}. The distance *d_S_* between two points in the camera coordinate system is compared with the distance *d_xy_* between two points in the world coordinate system. The comparative result determines whether the pair is desirable. Section 6.3 describes the concrete implementation. There are many symbols in the algorithm and their implications are as follows.

- f′*_i_* = (b′*_i_*, p′*_i_*): feature point *i* in the current image
- b′*_i_*: binary descriptor of feature point *i*
- p′*_i_*: 3D coordinate of feature point *i* in camera coordinate system
- F′ = {f′_1_, f′_2_, ⋯ , f′*_n_*}: current image feature point set
- f*_i_m__* = (b*_i_m__*, p*_i_m__*): feature point *i* in the map
- b*_i_m__*: the best match for *b_i_* and binary descriptor of feature point *i_m_*
- p*_i_m__*: 3D coordinate of feature point *i_m_* in world coordinate system
- F = {f_1*_m_*_, f_2*_m_*_, ⋯ , f*_n_m__*}: feature point set

In Algorithm 4, if |C| > *MIN_MATCH_SIZE*, we apply Algorithm 5 (three-point pose estimator+RANSAC) to C, if enough inliers are found then return “true”, otherwise return “false”. The algorithm is passed to a three-point pose algorithm plus RANSAC in order to perform a consensus and find the best camera pose that minimizes image distances in between camera projections of descriptors' 3D positions and their associated image coordinates. The pose with the biggest number of inliers is returned.


**Algorithm 4** multi feature point matching algorithm fusing local geometric constraints
**Input: F**′ = {**f**′_1_, **f**′_2_, ⋯ ,**f**′*_m_*}: current RGB-D image feature point set; *MIN_MATCH_SIZE*: minimum match points**Output:** C = {(**f***_i_*′, **f***_i_m__*)|**f***_i_* ∈ **F**′,**f***_i_m__* ∈ **F**}1:M = Φ, C = Φ ⊲ Set the initial value2:**for**
*i* = 1 → *n*
**do**3: Use hierarchical clustering to retrieve features, search the best match f*_i_m__* for f′*_i_*in feature point set F4: M = M ∪ {(f′*_i_*, f*_im_*)}5:**end for**6:Arbitrarily select matching point pair from M,c_a_ = (f′*_a_*, f*_a_m__*) ∈ M7:C = C U {c_a_}, M = M/{c_a_} ⊲ remove c*_a_* from M8:**if** |M| == 0 **then**9: **if** |C| > *MIN_MATCH_SIZE*
**then**10:  Call Algorithm 5 to pose estimate11: **end if**12:**end if**13:**while** |M|! = 0 **do**14: Arbitrarily select matching point pair from M,c*_b_* ∈ M15: *d_xy_*
**=** ‖p*_x_* − p*_y_*‖ ,*d_S_*
**=** ‖p′*_x_*
***−*** p′*_x_*‖16: **if** |*d_S_* − *d_xy_*| ≤ Δ*_d_*
**then**17:  C = C ∪ {c*_a_*}, M = M/{c*_a_*}18: **end if**19: **if** |C| > *MIN_MATCH_SIZE*
**then**20:  Call the Algorithm 5 to pose estimate21:**end if**22:**end while**


The pair c*_a_* = (f′*_a_*, f′*_am_*) ∈ M is precondition for finding a chain *C*. In order to increase the probability of satisfying the condition, it can be improved in two aspects. On the one hand, we select c*_a_* which has low similarity with other features from M. In the searching of the hierarchical clustering tree, we only record the number of its leaf nodes in each cluster node. We get the size of the hash bucket and the number of leaf nodes in each cluster and reduce them as much as possible. The feature point pairs we need are easy to be selected. On the other hand, we encounter this situation that the pairs in *M* are all tested but |*C*| < *MIN_MATCH_SIZE*, can choose another c*_a_* and try again with *C*.


**Algorithm 5** three-point pose estimator + RANSAC
**Input:** corresponding 3D point set {*p_i_*}, {*q_i_*}; the maximum distance: *d_max_*; the maximum number of iterations :max *Iterations***Output**: best pose estimation (R*, t*), Θ1:Θ = Φ, *c_max_* = 0, *Iterations* = 02:**while**
*Iterations* <= *maxIterations*
**do**3: Randomly select three pairs of corresponding points from {*p_i_*}and {*q_i_*}, use three-point method to compute *R* and *t*.4: Inliers=Φ5: *c_i_* = 06: **for**
*p_i_* , *q_i_* ∈ {*p_i_*}, {*q_i_*} **do**7:  **if** ‖*q_i_* − (*R* · *p_i_* + *t*)‖ < *d_max_*
**then**8:   *Inliers* = *Inliers* ∪ {*i*}9:   *c_i_* = *c_i_* + 110:  **end if**11: **end for**12: 
$C=\underset{i}{\text{∑}}\min\left(c_{i},\varsigma \right)$13: **if**
*C* > *C_max_*
**then**14:  Θ = *Inliers*15:  *C_max_* = *C*16: **end if**17:**end while**18:
$\left(R^{*},t^{*}\right)=\underset{R,t}{\arg\min}\underset{i\in \Theta }{\text{∑}}{\left\|q_{i}-\left(R\cdot p_{i}+t\right)\right\|}^{2}$19:**return** (R*, t*), Θ


### 6.3. Pose Estimation

Pose estimation is an important step in multi feature points matching. The method based on a three-point pose estimator plus RANSAC(RANdom SAmple Consensus) [39] is good pose estimation method. The basic idea of the method is to acquire feature point pairs from the color image, compute 3D coordinate of every feature point by the depth image and get two corresponding 3D point set {*p_i_*}, {*q_i_*}(*i* = 1, 2, ⋯ , *N*(*N* > 3)). If {*p_i_*} and {*q_i_*} satisfy rigid transformation relation, which is *q_i_* = *R* · *p_i_* + *t*, pose estimation module is used to solve *R*, *t*. The algorithm is shown in Algorithm 5.

In the algorithm, the computational formula of the total inliers is $C=\underset{i}{\text{∑}}\min\left(c_{i},\varsigma \right)$, *c_i_* represents the total inliers *c_i_* in *U_i_*(the i-th volume unit), *ς* represents the maximum limit of inliers counting in individual volume unit. We adopt RANSAC method to iterate and seek the right corresponding point set, then randomly select three pairs of corresponding points to calculate the transformation and use formula $C=\underset{i}{\text{∑}}\min\left(c_{i},\varsigma \right)$ to count inliers, finally select corresponding point set with the most inliers to compute rigid transformation.

After this, one of the graph pose optimization approaches [40] can then be used to reduce odometry errors using the computed transformations. In the following experiments, we adopt TORO(Tree-based netwORk Optimizer) [41] to do optimize the pose graph.

## 7. Experimental Results

We implemented the above algorithms and transplanted them into the RTAB-Map system [42]. In detail, we added FAST/BRAND as the ninth kind of feature extraction/description option of the system, added local geometric constraints option in the hypotheses verification, and added hierarchical clustering trees as one of the options of the nearest neighbor strategy. Since we lack a RGB-D dataset which have the ground truth of frame pairs, we can not directly evaluate our algorithms in terms of precision-recall metrics [16]. We finally evaluate our algorithms by comparing with the algorithms in RTAB-Map using one sequence in TUM RGB-D Dataset [43] and one captured in our lab, the former has the ground truth of trajectory and the latter is relatively longer and bigger. In the following experiments, unless special instructions, the RTAB-Map's default parameter values are used, and the BRAND descriptor size is set to 64, the BRAND degree threshold is set to 45 degrees. All the experiments are done using the same notebook computer, which has an Intel Core i7-4700HQ CPU and 8.0GB RAM with the Ubuntu 14.04 64bit operation system.

**Figure 4:**
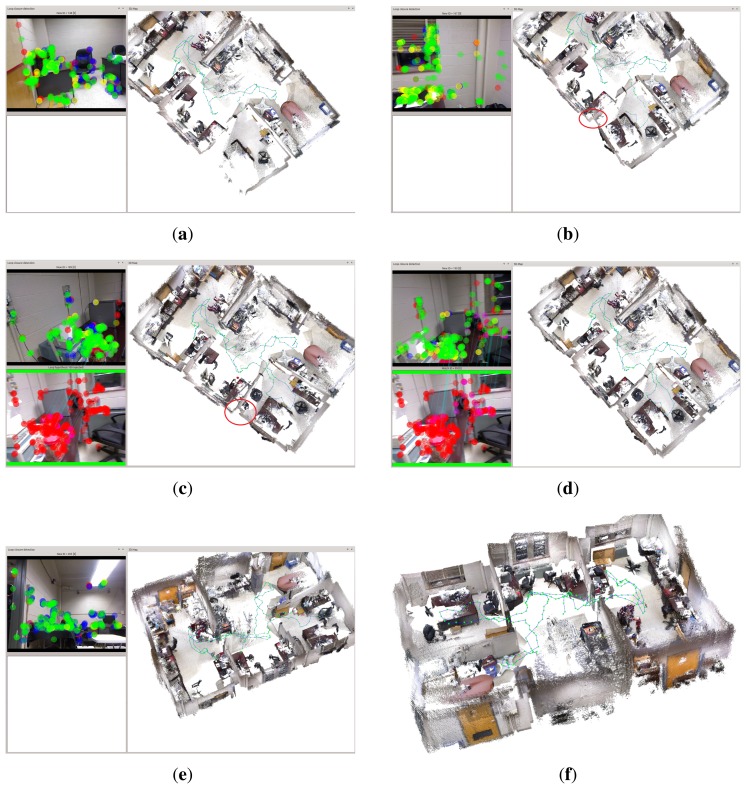
Experimental results of the sequence captured in our lab using our algorithms. (**a**) at 148 s; (**b**) at 167 s; (**c**) at 189 s; (**d**) at 190 s; (**e**) at 205 s; (**f**) the final map.

**Figure 5:**
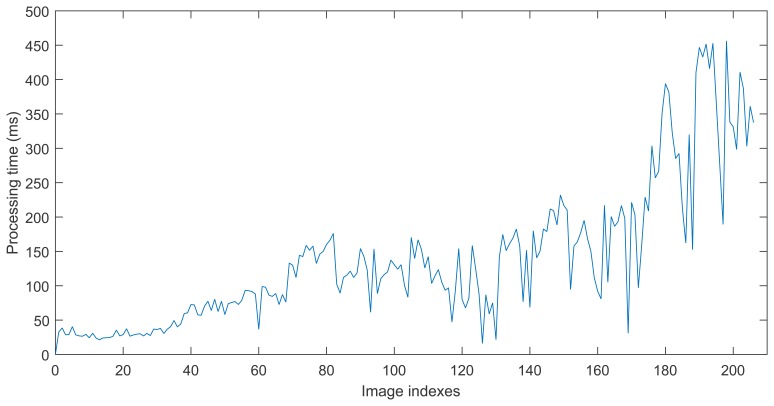
Total processing time of every key frame of the sequence captured in our lab using our approach.

The first experiment is to deal with a sequence captured in our lab using the proposed approach. Relevant parameter settings in Algorithm 4 are as follows: Δ*_D_* = 0.2 m, *MIN_MATCH_SIZE* = 20. Figure 4a–e shows one of the loop closing procedure and Figure 4f shows the final built 3D map. When considering Figure 4 with more attention, it may be observed that at 148 s (the loop closure detection frequency is 1 Hz), there are serious distortion and deformation with the built temp map (see Figure 4a). At 167 s, due to the distortion and deformation, serious dislocation appears in the red oval indication area(see Figure 4b). At 189 s, due to the successful detection of loop closure, after the graph optimization, the map is made a significant adjustment and dislocation which appears in the red oval indication area almost disappears. At 189 s, another loop closure is detected and accepted, and the map is updated accordingly. After that time, the distortion and deformation of the map almost disappear. Additionally, from Figure 4b we can see that the current frame and reference frame has a great difference of rotation and translation, and the light intensity is also changed, but our approach can correctly detect the loop closure while in the follow experiments this case is not detected in the other approaches in RTAB-Map. Timing performance of this experiment is shown in Figure 5.

**Figure 6:**
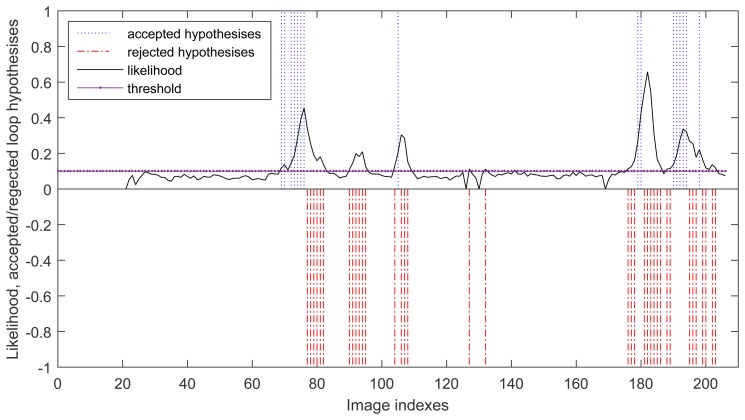
Loop closure detection for the sequence captured in our lab using our approach.

During the run, there are 52 cases where the probability is above the threshold but only 16 hypotheses are passed the local geometric constraint tests. The others are considered as wrong and they has been conveniently rejected by the geometric constraint test. This is shown in Figure 6, where the accepted loop closure hypotheses are set to be 1 and the rejected loop closure hypotheses are set to be −1 for clearly description.

**Figure 7:**
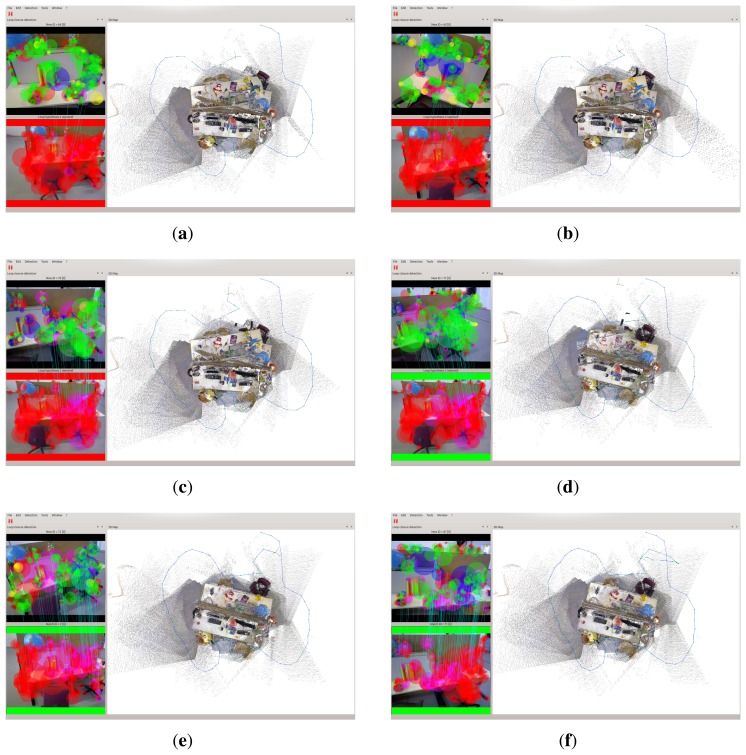
Experimental results of sequence “freiburg3_long_office_household”. (**a**) at 66 s; (**b**) at 68 s; (**c**) at 70 s; (**d**) at 71 s; (**e**) at 72 s; (**f**) at 81 s.

The second experiment is to deal with the sequence “freiburg3_long_office_household” of TUM RGB-D Dataset using the proposed algorithms and the algorithms designed in RTAB-Map. At this time, we set the nearest neighbor strategy to Linear for all algorithms. All the algorithms can successfully complete the SLAM procedure in this experiment except using FAST/FREAK as detector/descriptor. Figure 7 shows the loop closing process of using our algorithms. At 71 s, the first accepted loop closure is detected and the map is corrected accordingly, which can be seen from the desks' changes in the map before and after this time point. Figure 7f shows the final mapping result using our algorithms (FAST/BRAND + local geometric constraints). Table 1 shows the comparing results of our algorithms with the algorithms in RTAB-Map. As shown in Table 1, using FAST/BRAND can get more accepted loop closures, which proves that adding depth geometric information in the descriptor does increase the matching accuracy, and consequently increase the mapping accuracy. Since this dataset has ground truth of trajectory, we use the absolute trajectory error (ATE) evaluation tool provided with the benchmark to calculate the root mean squared error (RMSE) of the key frame pose errors summed over the entire trajectory. From Table 1, we can see that using FAST/BRAND can get the most accurate pose estimate.

The third experiment is to deal with the sequence used in the first experiment, but uses the algorithms and parameters set as same as those in the second experiment. The loop closure detection results are summarized in Table 2. As shown in Table 2, there are three kinds of algorithms cannot handle this sequence, and using FAST/BRAND can get more accepted loop closures.

## 8. Conclusions and Future Works

In this paper, we have presented multi feature points matching fusing local geometric constraints for performing loop closure detection under aliasing conditions. Feature is extracted from RGB-D image and described by binary descriptor. At the searching stage of the algorithm, we employ the locality-sensitive hashing method and hierarchical clustering based searching algorithm for a fast search. In order to avoid as much false loop closing information as much as possible, this paper focus on multi feature points matching. We demonstrate the quality of our approach with results obtained when deal with the sequence captured in our lab and the sequences from the benchmark dataset.

Future work concentrates on long image sequences in outdoor environment. Since outdoor environment is very complicated, the algorithm will be affected by many factors. The system put in place in this paper is conducted as the basis in improving the robustness of SLAM systems in handling large loop. The algorithm will be extended to larger environments to retain high accuracy and speed.

## Figures and Tables

**Table 1:** Comparing results of dealing with the sequence “freiburg3_long_office_household”.

**Detector/Descriptor**	**Loop Accepted**	**Loop Rejected**	**RMSE (m)**
**SURF**	4	12	0.064
**ORB**	0	11	0.562
**SIFT**	3	16	0.062
**FAST/FREAK**	–	–	–
**FAST/BRIEF**	6	14	0.058
**GFTT/FREAK**	6	14	0.061
**GFTT/BRIEF**	3	16	0.069
**BRISK**	6	13	0.055
**FAST/BRAND**	9	14	0.033

**Table 2:** Comparing results of dealing with the sequence captured in lab.

**Detector/Descriptor**	**Loop Accepted**	**Loop Rejected**
**SURF**	8	37
**ORB**	–	–
**SIFT**	12	36
**FAST/FREAK**	–	–
**FAST/BRIEF**	11	36
**GFTT/FREAK**	–	–
**GFTT/BRIEF**	9	33
**BRISK**	10	31
**FAST/BRAND**	16	36
